# Beneficial effects on host energy metabolism of short-chain fatty acids and vitamins produced by commensal and probiotic bacteria

**DOI:** 10.1186/s12934-017-0691-z

**Published:** 2017-05-08

**Authors:** Jean Guy LeBlanc, Florian Chain, Rebeca Martín, Luis G. Bermúdez-Humarán, Stéphanie Courau, Philippe Langella

**Affiliations:** 1 Centro de Referencia para Lactobacilos (CERELA-CONICET), San Miguel de Tucumán, Argentina; 2 Commensals and Probiotics-Host Interactions Laboratory, Micalis Institute, INRA, AgroParisTech, Université Paris-Saclay, 78350 Jouy-en-Josas, France; 3 Merck-Médication Familiale, BP 77035, 21070 Dijon, France

**Keywords:** Microbiota, Vitamins, Short-chain fatty acids, Energy metabolism

## Abstract

The aim of this review is to summarize the effect in host energy metabolism of the production of B group vitamins and short chain fatty acids (SCFA) by commensal, food-grade and probiotic bacteria, which are also actors of the mammalian nutrition. The mechanisms of how these microbial end products, produced by these bacterial strains, act on energy metabolism will be discussed. We will show that these vitamins and SCFA producing bacteria could be used as tools to recover energy intakes by either optimizing ATP production from foods or by the fermentation of certain fibers in the gastrointestinal tract (GIT). Original data are also presented in this work where SCFA (acetate, butyrate and propionate) and B group vitamins (riboflavin, folate and thiamine) production was determined for selected probiotic bacteria.

## Background

It has been well established that adenosine triphosphate (ATP) plays a crucial role in cell biology since it transports within the cells the chemical energy required for numerous metabolic processes. When an energy demanding process is required, ATP is converted to its precursor adenosine diphosphate or adenosine monophosphate, and when involved in energy producing reactions, these precursors are recycled back to ATP as energy storage units. Needless to say, the human body obtains its energy from foods that contain carbohydrates, proteins and fatty acids which are used in different metabolic reactions in order to increase cellular ATP levels which are essential for life itself.

Aerobic organisms, such as humans, use the Krebs cycle (also known as the citric acid cycle or the tricarboxylic acid cycle), which is a series of chemical reactions, to obtain energy from either the metabolisms of glucose and/or amino acids or the degradation of fatty acids. These energy producing processes require the use of different compounds that should also be obtained by exogenous sources (food) such as Short Chain Fatty Acids (SCFA) and certain B group vitamins.

In this review, we will describe the potential beneficial roles of SCFA and vitamins produced by commensal, food-grade and probiotic bacteria. We will particularly emphasize the impact of these bacterial products on host energy metabolism and consecutively on fatigue. Our hypothesis is that a better regulation of the production of these energetic metabolites by these bacteria could help to salvage energy.

## The human gut microbiota and host energy metabolism

The human gut microbiota plays a major role in the direct ingestion of foods but also in the mammalian nutrition system. Most of the absorption and digestion of ingested food takes place in the gastrointestinal tract (GIT) where the commensal bacteria, components of the gut microbiota, play a very important role. One of the first described contributions of the commensal bacteria to human metabolism and physiology was their ability to produce vitamin B12 [1]. Afterwards, it was shown that they can also synthesize vitamins B and K [2]. They also play a role in proteins, lipids and more importantly in carbohydrates metabolism [3]. These commensal bacteria ferment carbohydrates, principally non-digestible carbohydrates that are not used by the host, into CO_2_, H_2_ and CH_4_ and short-chain fatty acids (SCFAs) primarily acetate, propionate and butyrate [4]. Most of these SCFAs produced in the intestine are then absorbed by the host and contribute to its energy.

The commensal bacteria can also transform some plant-derived non-nutritional substances, such as flavonoids, leading to the formation of a large variety of nutritional metabolites for humans [5]. In fact, the human gut microbiota influences the systemic metabolism of the host, modulating the metabolic profile of topographically remote organs such as the liver and the kidney [6].

Moreover, besides the commensal bacteria, transiting food-grade and probiotic bacteria are also playing a role in host energy metabolism through the production of some biogenic compounds in functional foods: they (i) improve the nutritional composition of these foods in free vitamins, bioactive peptides and γ-amino butyric acid (GABA) [7–9] and (ii) increase the concentration of free amino acids and other nutritional compounds and metabolites [10–12]. All these commensal, food-grade and probiotic bacteria are interacting with the host cells which absorb nutrients, water and electrolytes [13]. They are contributing to energy production through glucose synthesis and degradation: neoglucogenesis and glycogenolysis. Neoglucogenesis is a ubiquitous process that results in the synthesis of glucose from non-carbohydrate carbon substrates such as pyruvate, lactate, glycerol, glucogenic amino acids, and fatty acids. Neoglucogenesis and the glycogenolysis (degradation of glycogen) are the main mechanisms in humans to maintain an appropriate level of glucose, the most important energy source for humans.

## The role of short chain fatty acids (SCFAs) produced by commensal and probiotic bacteria in host energy intake

### SCFAs produced by commensal bacteria

The non-digestible carbohydrates, including cellulose, xylans, resistant starch and inulin, are fermented in the colon by the anaerobic colonic bacteria to yield energy for microbial growth and end products such as SCFAs [14]. SCFAs have been shown to exert many positive effects on mammalian energy metabolism. In addition to glucose, mammals utilize these SCFAs (also named volatile fatty acids) as a metabolic fuel [15]. SCFAs, mainly acetate, propionate and butyrate [16], are essential for the health and wellbeing of the host when present in sufficient quantities. Moreover, the presence of carbohydrates (dietary fibers, prebiotics) is essential to orientate the metabolic activity in the direction of carbohydrates fermentation [17]. In fact, it has been demonstrated that 70% of the energy obtained by intestinal epithelial cells (IECs) is derived from butyrate which is mainly produced by commensal bacteria especially Clostridia species belonging to Firmicutes such as *Ruminococcus* and *Faecalibacterium* [18] (see Table 1).

**Table 1:** Vitamin and short chain fatty acids (SCFA) producing bacteria

Microorganism/s	Type	Compound	References
*Ruminococcus, Faecalibacterium*	Commensal	Butyrate	[18]
Bifidobacteria	Probiotic	Acetate/lactate	[27]
*L. salivarius* spp *salcinius* JCM 1230 *L. agilis* JCM 1048	Probiotic	Propionate/butyrate	[29]
*L. acidophilus* CRL 1014	Probiotic	Acetate/butyrate/propionate	[30, 33–35]
LGG	Probiotic	Propionate	This study
*B. longum* SP 07/3	Probiotic	Propionate/acetate	
*B bifidum* MF 20/5	Probiotic	Propionate/acetate	
*L. gasseri* PA 16/8	Probiotic	Propionate	
*L. plantarum* WCSF1	Comensal	Folate	[46]
Bifidobacteria	Food-grade	Thiamin	[50, 51]
*Lactococcus, Leuconostoc*	Food-grade	Thiamin	[52]
*L. sanfranciscensis*	Food-grade	Thiamine	[53]
*L. lactis*	Food-grade	Riboflavin	[60]
*L. fermentum,*	Food-grade	Riboflavin	[61]
*Leuconostoc mesenteroides* and *Propionibacterium freudenreichii*	Food-grade	Riboflavin	[62–65]
*L. plantarum*	Food-grade	Riboflavin	[41]
151 LAB strains	Food-grade	Folate	[40]
40 LAB strains	Food-grade	Folate	[43]
36 LAB strains	Food-grade	Folate	[42]
*L. fermentum* CECT 5716	Probiotic	Vit B2 and B9	[38]
LGG	Probiotic	Vit B1, B2 and B9	This study
*B adolescentis* DSM 18350	Probiotic	Folate	[83]

(Non exhaustive list)

SCFAs have been pointed out as the link between diet, gut microbiota, and host energy metabolism [19]. It has been estimated that when taken up, a large part of the SCFAs is used as a source of energy and this could provide nearly 10% of our daily caloric requirements [20]. Recently, studies with labeled SCFAs infused in mice have shown that 62% of infused propionate were used as gluconeogenic substrate in whole body glucose production. Glucose synthesis from propionate accounted for 69% of total glucose production and the synthesis of palmitate and cholesterol in the liver from caecal acetate and butyrate as substrates while synthesis from propionate was low or absent [21]. All these data support the fact that SCFAs (acetate, propionate, and butyrate) produced by the human gut microbiota are playing important roles as substrates for glucose, cholesterol, and lipids metabolism. Butyrate is the energy substrate for the colonic epithelium and acetate and propionate for peripheral tissues [19]. To better understand the relationship between SCFAs and host energy metabolism, we need to decipher the SCFAs pathway and signaling, focusing on different free fatty acid (FFA) receptors. For instance, two FFA receptors, GPR43 and GPR41, have been very recently reported to regulate host energy homeostasis in the GIT and adipose tissues [15, 22]. GPR43-deficient mice are obese on a normal diet, whereas mice overexpressing GPR43 specifically in adipose tissue remain lean even when fed with a high-fat diet [23].

The impact of all these relationships is more evident when the intestinal homeostasis is broken. As the gut microbiota affects nutrient acquisition and energy regulation of the host, it can influence the development of obesity, insulin resistance, and diabetes [22]. In fact, obesity and type 2-diabetes mellitus are characterized by a lower abundance of specific bacteria (such as *Akkermansia muciniphila* and others) and SCFAs leading to gut-barrier dysfunction, low-grade inflammation and altered glucose, lipid and energy homeostasis [24]. SCFAs are also playing important roles in inflammation and cancer. It is thus interesting to mention that *Faecalibacterium prausnitzii*, the first anti-inflammatory commensal bacterium identified on the basis of human clinical data, is also one of the major butyrate-producer of the human intestinal microbiota [25, 26].

### SCFAs produced by probiotic bacteria

According to the WHO/FAO, probiotics are “live microorganisms that, when administered in adequate amounts, confer a health benefit on the host”. In this sense, the most commonly used probiotics are lactic acid bacteria (LAB) and bifidobacteria. Lactobacilli can produce SCFAs (i) by the fermentation of carbohydrates end-products such as pyruvate, which is generated during the glycolytic pathway; and also (ii) by the phosphoketolase route in the heterofermenting conditions [27]. Bifidobacteria are using the fermentation pathway to produce mainly acetate and formate during growth under carbohydrates limitation, and acetate and lactate when carbohydrates are in excess [28]. *In vivo*, acetate enters the peripheral circulation to be metabolized by muscles and other tissues, while propionate is taken up by the liver [27]. This ability to produce SCFAs by both lactobacilli and bifidobacteria is highlighted when the SCFAs concentration is analyzed under different microbiota compositions. For instance, supplementation with *Lactobacillus salivarius* ssp. *salicinius* JCM 1230 and *L. agilis* JCM 1048 during 24 h in a simulated chicken cecum was shown to significantly increase propionate and butyrate formation [29]. *L. acidophilus* CRL 1014 was also recently shown to increase SCFAs concentration in SHIME (for Simulator of Human Microbial Ecosystem) reactor [30].

Growth and metabolic activity of probiotic bacteria such as bifidobacteria and lactobacilli, can be selectively stimulated by various dietary carbohydrates not digested by the host, called “prebiotics”. In fact, the combination probiotics-prebiotics (called synbiotic) is able to shift the predominant bacteria and the production of SCFAs of fecal microbiota in a model system of the human colon [31]. The production of SCFAs by these bacteria is potentially an essential regulatory effector of epithelial proliferation in the gut [32].

One of the best-characterized probiotic strain, *Lactobacillus rhamnosus* strain GG (LGG), has been included in several studies with mix of probiotic strains and prebiotics where it was able to metabolize these prebiotics leading to SCFAs production.

**Fig. 1:**
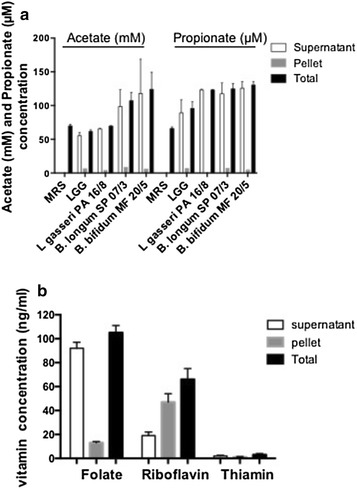
**a** Acetate and propionate production by *Lactobacillus rhamnosus* GG and *L. gasseri* PA 16/8, *Bifidobacterium longum* SP 07/3 and *B. bifidum* MF 20/5in supernatant (*white bars*), cellular extracts (*stripped bars*) and total production (*black bars*). The bacterial strains have been grown overnight in MRS medium (plus cysteine for bifidobacteria strains) at 37 °C. Cultures were then centrifuged (5000*g* for 10 mn at 4 °C). Supernatants and pellets were separated and frozen in liquid nitrogen immediately. The error bars are SEM (Standard Error Mean) and the experiments were performed four times. They were kept at −80 °C until further analyses. Acetate, butyrate and propionate were quantified in supernatant and pellets by Mass Spectromectry. **b** B group vitamin production by *L. rhamnosus* GG in supernatant (*white bars*), cellular extracts (*stripped bars*) and total production (*black bars*). The probiotic strain was grown in folate, riboflavin or thiamin free media (Difco) after which cells were centrifuged (4000×*g*) and washed with saline solution (0.85% NaCl, m/v). Folates and riboflavin were quantified according to previously described microbiological methods [41, 88] and thiamin using a Xevo Triple-Quadrupole mass spectrometer (Waters Corporation) equipped with an electrospray ionization interface coupled to an Acquity H-Class UPLCTM device (Waters Corporation) according to Waters application notes LGC/R/2011/181

The few available human clinical studies based on the production of SCFAs have been performed using a mixture of probiotics and prebiotics. Note that no human clinical studies have been performed using any probiotic strain alone such as LGG in the field of SCFAs production. Some bifidobacteria strains have been characterized in terms of acetate and lactate production [33–35]. Here, we have thus evaluated the in vitro potential to produce and release *de novo* SCFAs of four probiotic bacterial strains: LGG; *Bifidobacterium longum* SP 07/3, *B. bifidum* MF 20/5 and *L. gasseri* PA 16/8. LGG was able to produce and release propionate in a significant amount (89 µM of propionate in MRS medium), but was not able to produce either butyrate or acetate (Fig. 1a). *B. longum* SP 07/3and *B. bifidum* MF 20/5 were able to produce and release acetate in a significant amount. *B. longum* SP 07/3*, B. bifidum* MF 20/5 and *L. gasseri* PA 16/8were able to produce and release propionate in a significant amount, but were not able to produce butyrate (Fig. 1).

SCFAs can exert multiple beneficial effects on various aspects of mammalian energy metabolism; however, our understanding of the underlying molecular mechanisms remains incomplete. This situation is partly due to the lack of human data since most of the results were obtained in rodents and cannot be directly translated to humans. Moreover, the field is severely hampered by the lack of data on actual fluxes of SCFAs and metabolic processes regulated by SCFAs. Most studies report concentrations of metabolites (fatty acids, glucose, cholesterol, etc.) or transcript levels, but these do not necessarily reflect flux changes.

A number of questions need to be addressed: (1) what are the in vivo SCFAs production and uptake fluxes under different conditions (i.e., with different fibers, with different microbiota, or in different disease models)? (2) How do these SCFAs then affect glucose and lipids fluxes via their dual role as substrates and regulators? And (3) does the demand of the host for specific SCFAs drive a change in microbial metabolism?

A quantitative and time-resolved approach to these questions should bring a great step forward to elucidate the role of SCFAs in mammalian energy metabolism. In this regard, Van den Abbeele et al. [36] have shown the potential role of some commensal bacteria in the production of SCFAs [32]. Based on this scheme, we can consider that diet supplement containing probiotic lactobacilli and/or bifidobacteria can probably contribute positively to this process and thus play a role in this process.

## The key role of vitamins produced by commensal and probiotic bacteria in host energy metabolism

**Fig. 2:**
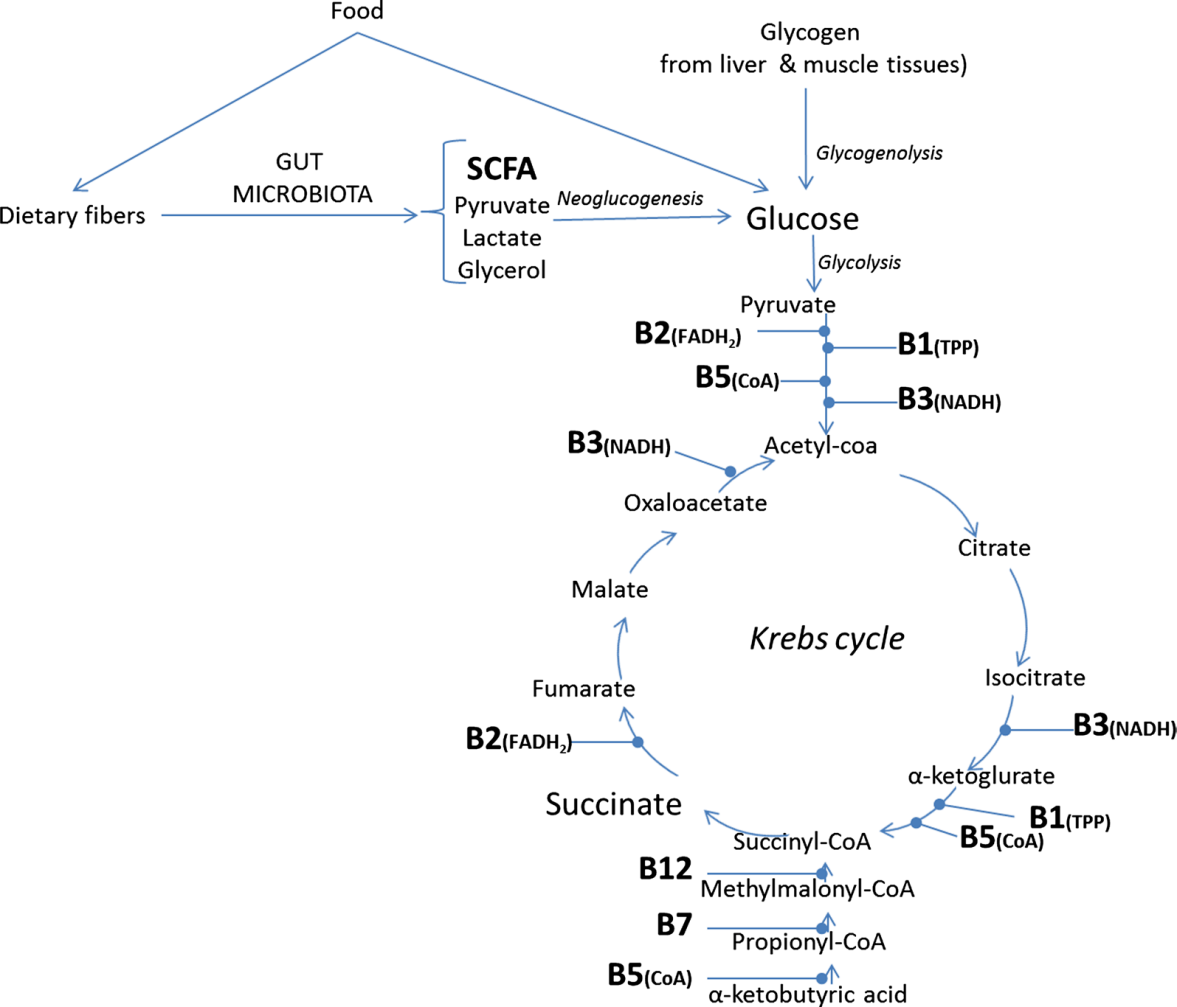
Whereare microbial synthesized short-chain fatty acids (*SCFA*) and B group vitamins (*B1* thiamin, *B2* riboflavin, *B3* niacin, *B5* panthothenic acid, *B7* biotin, *B12* cobalamin) involved in energy metabolism? In parenthesis are the active forms of the co-factors involved in each reaction (FADH2 flavin adenine dinucleotide in hydroquinone form, *CoA* acetyl coenzyme A, *NADH* nicotinamide adenine dinucleotide, *TPP* thiamin pyrophosphate)

As previously stated in the introduction, to convert food into ATP, the energy storing molecule, numerous co-factors including B group vitamins are involved. Although most of these vitamins are not essential for each metabolic reaction, they are cofactors of enzymes that act as catalyzers so that the reactions can occur at a high enough rate to produce energy at a rate compatible with life. Most B group vitamins are directly involved in energy metabolism and these functions. In order to facilitate where each vitamin acts, a schematic review of energy metabolism is provided in Fig. 2.

Thiamin (vitamin B1), as thiamine diphosphate (TPP), plays a fundamental role in host energy metabolism since it acts as a co-factor for enzymatic reactions that cleaves α-keto acids such as pyruvic acid [37]. The role of riboflavin in energy metabolism is even more evident since it is phosphorylated into Flavin Adenine Dinucleotide (FAD) and acts as oxidative agents through its capacity to accept a pair of hydrogen atoms. It then catalyzes the decarboxylation of pyruvate to acetyl-CoA and the conversion of α-ketoglutarate to succinyl-CoA which is the 5th reaction of the Krebs cycle. Niacin (vitamin B3) in the form of Nicotinamide Adenine Dinucleotide (NAD) is the electron acceptor for isocitrate dehydrogenase, α-ketoglutarate dehydrogenase and malate dehydrogenase. Pantothenic acid (vitamin B5) is required for synthesis of coenzyme A (CoA) required for the pyruvate dehydrogenase complex, α-ketoglutarate dehydrogenase, and branched-chain α-ketoacid dehydrogenase. During the catabolism of fatty acids with an odd number of carbon atoms and certain amino acids (valine, isoleucine, methionine, and threonine), propionyl-CoA is converted to succinyl-CoA for oxidation in the Krebs cycle through enzymes that requires vitamin B12 (cobalamin) or vitamin B7 (biotin) as co-factors.

### Vitamins production by commensal, food-grade and probiotic bacteria

Although most LAB and bifidobacteria are considered auxotroph for vitamins production, there is an increasing amount of evidence that certain strains of these two groups of bacteria can produce B-group vitamins. Microorganisms isolated from a variety of ecological niches such as the GIT of humans and other animals [2, 38, 39], dairy products [40–42], plants [43] and grains [43], have been shown thus to produce varying amounts of vitamins B1, B2, B12 or B9. The genetic and biochemical pathways of these B group vitamins biosynthesis is well known [2] and will not be discussed in this review, but it is important to state that regardless of their origin, certain strains can produce elevated concentrations of vitamins. These vitamins are normally stored inside the cells and released by direct diffusion, using specific transporters in the cell membrane or via cellular lysis either in their growth media or inside the GIT of the host, making these strains ideal candidates for the in situ delivery of B group vitamins.

#### Vitamins production by commensal bacteria

Although it has been suggested that some commensal bacterial species can synthesize essential vitamins, especially of the B and K groups [44], there are very few works that have been able to isolate and prove that specific commensal bacteria are able to produce these vitamins and no data on their concentrations effectively produced in the GIT have been published yet. In this sense, it has been shown that *L. plantarum* WCSF1, a commensal *Lactobacillus* strain isolated from human saliva, was shown to possess the folate biosynthesis genes in its genome [45] and that this strain can produce folate in culture media [46]. However, it was shown that only one genetic modification allowed this strain to produce enough of the vitamin to generate a methotrexate (a folate antagonist) resistant phenotype that is observed in high-folate producing strains [47]. Also, it was shown that the riboflavin biosynthesis operon has been shown to be interrupted in *L. plantarum* WCFS1 genome resulting in its inability to produce riboflavin or other vitamins [45].

In a genome assessment of 256 human gut bacteria, it was shown that 40–65% possessed the biosynthesis pathways for eight B-vitamins (biotin, cobalamin, folate, niacin, pantothenate, pyridoxine, riboflavin, and thiamin). However, all of the strains were not able to produce these vitamins in culture media [48]. These authors have hypothesized on the amounts of vitamins that certain strains could produce inside the GIT. However, these values are a very weak estimation since they are based on intracellular vitamin concentrations from other works where the strains were grown in laboratory conditions and do not reflect the hostile environment and substrate availability of the GIT.

The effects of vitamins produced by commensal bacteria need to be studied further, especially the amounts of vitamins produced in the GIT. Besides their nutritional/physiological properties, many of these vitamins have also been shown to be involved in the development and function of immune cells of the host since there is a direct link between commensal bacteria-derived vitamin biosynthetic intermediates and immune cells that directly recognize these intermediates [49].

#### Vitamins production by food-grade bacteria

There are only a few studies that have shown that LAB or other food-grade microorganisms have the capacity to produce thiamin. Strains of bifidobacteria were able to produce elevated concentrations of thiamin in soymilk and fermented milks [50, 51] as could the mesophilic starter cultures consisting of strains of *Lactococcus* and *Leuconostoc* [52]. *Lactobacillus sanfranciscensis* isolated from fermented cereals was capable of producing thiamine [53] and in a screening trial of 83 LAB strains isolated from fermented pickles, 50 were able to grow in thiamin free culture media but only produced very low concentrations of the vitamin [54]. In this sense, it was also shown that *L. salivarius* CRL1328 did not require thiamin for its growth [55]. The biosynthesis of thiamin in prokaryotes was described in detail for *Escherichia coli, Salmonella typhimurium* and *Bacillus subtilis* and *L. reuteri* ATCC 55,730 [56, 57]. In a recent study, *L. plantarum* WCFS1 was shown to be able to produce thiamin although some of the biosynthesis genes were missing [48]. The metabolic pathways of this latter strain were further studied and for thiamin biosynthesis, initially 3 of 10 required reactions were not coupled to a gene. However, it was suggested that there are orthologs such as *MoaD* and *MoeE*in the *L. plantarum* genome which could be involved in thiamin biosynthesis [58] and could explain its thiamin producing capability.

As it was the case for thiamin, there are limited studies that have shown that LAB or other food-grade bacteria can produce riboflavin, although the number of B2 producing strains is significantly higher. As example, it was shown that 42 strains of LAB isolated from a variety of fermented dairy products were able to produce riboflavin [41] as did 8 strains from goat milk and cheeses [40]. Moreover, roseoflavin has been used to obtain constitutive riboflavin overproducing strains of *B. subtilis* [59], *Lactococcus lactis* [60], *L. plantarum* [41], *L. fermentum* [61] *Leuconostoc mesenteroides* and *Propionibacterium freudenreichii* [62–65]. Some of these have been shown to provide beneficial effects in vitamin depleted animals and could be inserted as novel starter cultures [66–69].

The role of folates in energy metabolism is not as direct as thiamin and riboflavin. Folate dependent enzymes are involved in the metabolism of several amino acids including methionine. The synthesis of this important amino acid is catalyzed by methionine synthase, an enzyme that does not only require folate (as 5-methyltetrahydrofolate) but also vitamin B12. One of the most important roles of folate is its involvement in the methylation cycle. In terms of energy production, the methylation cycle serves to degrade excess methionine in the liver to homo-cysteine, which can either be catabolized to sulfate and pyruvate with the latter being used for energy in the Krebs cycle. Because LAB produce folate, there has been great interest to search folate producing strains as an alternative of the use of the chemically synthesized folic acid that is normally used in fortification programs and as dietary supplements that has been show to induce adverse side effects when consumed in large quantities [70]. A few recent examples of folate producing LAB are 151 strains isolated from goat milk and chesses [40], 40 from raw cereal materials [43], 36 strains from yogurt or cheese starter cultures [42], 25 from amaranth and 15 from quinoa (personal data). As is the case with all vitamins, production by LAB and bifodobacteria is a strain dependent trait. There are also reports of genetically modified strains that produce elevated concentrations of folates [71–74] and some of which have been shown to be effective in the reversion of folate deficiencies in mice [75, 76].

#### Vitamins production by probiotic bacteria

Although most vitamin producing-microorganisms identified so far cannot be considered probiotics because they lack essential studies (survival in the GIT, adherence to mucosal cells, evidence of their effects in humans clinical trials), there are promising probiotic strains that have been shown to be able to produce B group vitamins.

*L. fermentum* CECT5716 was originally isolated from human milk of healthy mothers [77] and the complete genome of this strain was sequenced [78]. There are 3 clinical studies published using this strain which were performed to evaluate their safety [79], immune-modulating properties [80], and capacity to prevent gastrointestinal and respiratory infections in infants [81]. This strain was able to produce both vitamins B2 and B9 [38] and the gene clusters responsible for the production of both vitamins were also identified. The production of vitamins has only been demonstrated in microbial culture media and none of the clinical trials evaluated serum vitamin concentrations of patients that received this probiotic.

*L. rhamnosus* GG, isolated from the GIT of a healthy human, is able to synthetize B1, B2 and B9 in culture medium (Fig. 1b). Although the synthesis levels observed seem to be low, animal or clinical trials should be performed to evaluate its effectiveness in improving the vitamin status of consumers. This is the only one probiotic strain where thiamin production has been demonstrated, making it an ideal candidate to increase energy metabolism of consumers.

*Bifidobacterium lactis* BB12, isolated from dairy products, is the most documented probiotic since it is described in 300 scientific publications out of which more than 130 are publications of human clinical studies [82]. According to the publically available genome sequence, it possesses all the genes for B1 biosynthesis but not for B2, B6, nor B9 (search in http://www.genome.jp/kegg/kegg2.html consulted on September 30th, 2016). However, there are currently no published studies to confirm this potential vitamin production by this strain.

*B. adolescentis* DSM 18350 is the first probiotic able to increase folate concentrations in humans [83]. In this pioneer study, a significant increase in fecal folate concentrations was observed in 13 volunteers who consumed 5 × 10^9^ CFU of the strain per day during 30 days. Since it is assumed that at least a portion of the folates produced are absorbed by the host, the fecal concentration would not strictly correlate with the total amount of *de novo* synthesized folates. Further studies need to be conducted in order to evaluate the effect of this strain on vitamins concentration in serum and red blood cells. This strain was first shown to be able to produce folate in culture medium [84] and then shown to enhance the folate status (increased plasma and liver concentrations) in rats [85] showing that animals studies can be a good indicator for beneficial effects in humans.

From a biochemical point of view, it is clear that these B-group vitamins are either directly or indirectly involved in energy metabolism and since certain LAB and bifidobacteria strains can produce these vitamins in very large amounts, there is an increasing interest in using such bacteria for the development of novel energy drinks or food supplements.

We have evaluated the in vitro potential to produce and release *de novo* vitamins B1/B2 and B9 of four probiotic bacterial strains: *L. rhamnosus* GG, *B. longum* SP 07/3, *B. bifidum* MF 20/5 and *L. gasseri* PA 16/8. *L. rhamnosus* GG was a good folate (B9) and riboflavin (B2) producer and releaser and a low, but significant, producer of intracellular thiamin without extracellular synthesis (Fig. 1b).

*B. longum* and *B. bifidum* were low but significant producer of intracellular thiamine (B1) without extracellular synthesis, but were not able to produce either folates (B9) either riboflavin (B2).

## Discussion and conclusions

The scientific data summarized in this review indicate a relationship between SCFA and B group vitamins produced by commensal and probiotic bacteria and energy metabolism by the host.

We propose that SCFAs and B group producing bacteria can increase the production of ATP; however, their direct impact on fatigue in human must be further evaluated to understand the relationship between fatigue and intestinal microbiota.

Recent studies conducted on chronic fatigue syndrome have already suggested a role for altered intestinal microbiota in the pathogenesis of this disease [86, 87] and therapeutic efforts to modify gut microbiota could be a means to modulate the development and/or progression of this disorder [87].

A new approach could be to evaluate the relationship between the ability of selected probiotics strains to produce energy metabolites and their impact on fatigue in humans. In order to evaluate the potential role of such metabolites in the prevention and recuperation of fatigue, further clinical trials are needed to (i) to determine the level of production of such metabolites in the gut after intake of selected probiotics strains, and (ii) assess and evaluate their impact on fatigue.
